# Dry anophthalmic socket syndrome – morphological alterations in meibomian glands

**DOI:** 10.1038/s41433-021-01426-z

**Published:** 2021-02-09

**Authors:** Alexander C. Rokohl, Marc Trester, Parsa Naderi, Niklas Loreck, Sarah Zwingelberg, Franziska Bucher, Keith R. Pine, Ludwig M. Heindl

**Affiliations:** 1 Department of Ophthalmology, University of Cologne, Faculty of Medicine and University Hospital of Cologne, Cologne, Germany; 2 Trester-Institute for Ocular Prosthetics and Artificial Eyes, Cologne, Germany; 3 UCL Institute of Ophthalmology, University College London, London, Great Britain; 4 School of Optometry and Vision Science, University of Auckland, Auckland, New Zealand; 5 Center for Integrated Oncology (CIO) Aachen-Bonn-Cologne-Düsseldorf, Cologne, Germany

**Keywords:** Inflammation, Conjunctival diseases, Eyelid diseases

## Abstract

**Purpose:**

To evaluate morphological alterations of meibomian glands (MGs) in the dry anophthalmic socket syndrome (DASS).

**Methods:**

Fifteen unilateral anophthalmic patients wearing cryolite glass prosthetic eyes were enrolled. All patients with clinical blepharitis or other significant eyelid abnormalities were excluded. In vivo laser scanning confocal microscopy (LSCM) of the MGs in the lower eyelids both on the anophthalmic side and the healthy fellow eye was performed to quantify acinar unit density, acinar unit diameter, acinar unit area, meibum secretion reflectivity, the inhomogeneous appearance of the glandular interstice, and inhomogeneous appearance of the acinar walls.

**Results:**

The lower eyelids of the anophthalmic sockets revealed a significant reduction of the acinar unit density (*p* = 0.003) as well as a significantly more inhomogeneous appearance of the periglandular interstices (*p* = 0.018) and the acinar unit walls (*p* = 0.015) than the healthy fellow eyelid. However, there were no significant differences regarding the acinar unit diameter, acinar unit area, and meibum secretion reflectivity of the MGs on the anophthalmic side compared to the healthy fellow eyelid (*p* ≥ 0.05, respectively).

**Conclusions:**

The eyelids of anophthalmic sockets without clinical blepharitis demonstrate a reduced density of MG acinar units and a more inhomogeneous appearance of the periglandular interstices and the acinar unit walls. This can cause meibomian gland dysfunction contributing to DASS and suggests early treatment of these symptomatic patients, even in the clinical absence of any blepharitis signs.

## Introduction

A majority of the anophthalmic patients suffer from dry anophthalmic socket syndrome (DASS) resulting in significant socket discomfort [1–35]. The degree of dry socket complaints ranges from trivial to severe and the reasons for DASS seem to be very variable, individual, and multifactorial [1–35]. Previous studies have reported dry eye symptoms in anophthalmic sockets and have investigated the reasons for that condition [17–26, 29, 30, 33, 35]. These studies showed partially reduced tear production and reduced reflex tears in anophthalmic sockets, in particular in symptomatic patients [18, 24, 26, 27, 29]. Other reasons for dry eye symptoms in anophthalmic sockets seem to include loss of goblet cells, lid margin abnormalities, lagophthalmos, a reduced tear meniscus, conjunctivitis, and eyelid laxity [21, 22, 24, 25, 27, 29, 33]. As a result, DASS has been defined as a disease of the socket surface characterized by a loss of tear film homeostasis accompanied by socket discomfort, in which tear film instability, conjunctival inflammation, and damage, as well as eyelid and neurosensory abnormalities, play etiological roles [35]. In addition, while a higher incidence of clinical blepharitis in anophthalmic sockets was reported in two small series [22, 29], it is not fully understood why over 63% of anophthalmic patients have significantly more subjective dryness complaints on the anophthalmic side compared to the healthy fellow eye, even in absence of tear deficiency and clinical blepharitis [35].

The authors are not aware of any systematic, prospective study investigating and quantifying morphological changes of meibomian glands (MGs) in anophthalmic sockets without signs of clinical blepharitis until now. We are also unaware of any study comparing the morphology of MGs of anophthalmic sockets with the healthy fellow eye in these patients [35]. Furthermore, the exact roles and interactions of etiological causes of DASS are obscure [35] and there is a high priority to establish a standardized examination protocol and to develop an evidence-based treatment algorithm for DASS [35].

The purposes of the present study were to evaluate potential morphological changes of MGs in anophthalmic sockets without clinical blepharitis using in vivo confocal laser scanning microscopy to compare the morphology of the MGs with the heathy fellow eye, to evaluate dry anophthalmic socket symptoms in these patients, and to investigate factors associated with potential morphological changes of the MGs.

## Subjects and methods

The study was approved by the Institutional Review Board of the University of Cologne and all performed procedures were in adherence to the tenets of the Declaration of Helsinki. Sixteen unilateral prosthetic eye patients of the Trester Institute, Cologne, Germany were approached over 11 consecutive days. Informed consent was obtained from all participants. Exclusion criteria included: not understanding the German language, younger than 18 years of age, less than 1 year wearing a prosthetic eye, a history of any ocular surface disease, known blepharitis or dysfunction of the MGs, surgical or laser interventions, contact lens wear, the use of topical medication, systemic diseases causing dry eye, chemotherapy, facial palsy, intravitreal operative injections, trigeminus or other facial nerve lesions, radiotherapy, eyelid injuries, socket or eyelid surgery in the last 3 months, and occlusion of the lacrimal system. Patients who used anti-inflammatory or antibiotic medication in either eye in the last half year were also excluded as were patients with defective or poor fitting prostheses.

Patients were asked face-to-face to complete a standardized dry eye questionnaire [36] modified for patients wearing prosthetic eyes. The first section asked demographic questions which included age, sex, ethnicity, date, the reason for eye loss, type of surgery, years of wearing a prosthesis, age of the present prosthesis, cleaning regime (>once daily, daily, between daily and weekly, weekly, between weekly and monthly, monthly, >monthly) and the presence of environmental factors causing dry eye. The second section focused on the history of their topical medication at any time point and external and systemic factors influencing dry eye symptoms. The third section included three established and standardized dry eye questionnaires [37–40]. Separately for the anophthalmic socket and the healthy fellow eye, German versions of the Ocular Surface Disease Index (OSDI), the 5-Item Dry Eye Questionnaire (DEQ-5), and the modified version of the Symptom Assessment iN Dry Eye (SANDE) questionnaire were completed. All vision-related questions were classified as not answered for the anophthalmic site.

A clinical examination was carried out to evaluate palpebral conjunctival inflammation using Pine et al.’s 0–4 grading scale [4, 5, 10] and the presence of eyelid abnormalities including ectropion, entropion, lagophthalmos, ptosis, as well as anterior and posterior blepharitis [35]. Schirmer I test (I-DEW Tearstrips, Mitron) following the application of one anesthetic eye drop (Oxybuprocaine 0.4%, Novesine^®^) was performed bilaterally [35]. The amount of wetting measured in millimeters was evaluated after 5 min [35]. Results under 5 mm were classified as pathological, 6–10 mm as borderline, and over 10 mm as normal [35].

### Image acquisition

In vivo laser scanning confocal microscopy (LSCM) was performed bilaterally with the Heidelberg Retina Tomograph III (Heidelberg Engineering GmbH, Dossenheim, Germany) using a diode laser with a wavelength of 670 nm. The HRT was equipped with the Rostock Cornea Module (RCM, 63x water-immersion objective lens, Carl Zeiss Meditec, Inc., Dublin, CA) enabling a scanning area of 400 × 400 µm with a resolution of 380 × 380 pixels. The RCM was covered with a sterile polymethacrylate cap (Tomo-Cap; Heidelberg Engineering). Before every examination session, one drop of Oxybuprocaine 0,4% (Novesine^®^) was instilled bilaterally. After the patients‘ head was placed in the headrest with the eyes looking at a fixed point slightly upwards, the lower eyelid was everted, and the centre of the Tomo-Cap was horizontally positioned on the centre of the lower eyelid margin. The focus was adjusted manually while the section mode imaging modality was used. Images of the meibomian acinar units were taken manually while the focus was continuously adjusted into deeper layers of tissue.

### Image analysis

**Fig. 1:**
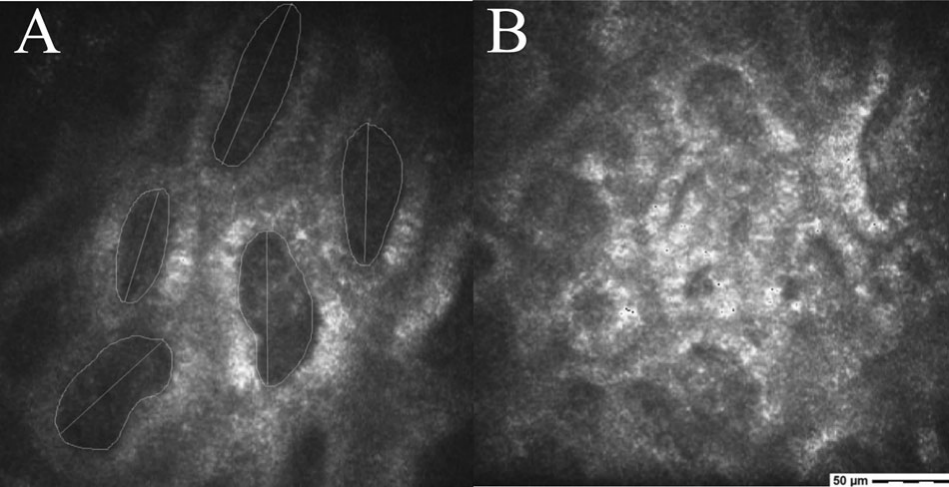
Confocal microscopy of the meibomian glands. Acinar units of the lower eyelid of a healthy fellow site including area and longest diameter measurements (**A**). The acinar unit walls were clearly definable, have relatively low reflectivity, and the interstice had a very homogeneous appearance (**A**) compared to the anophthalmic site with a very inhomogeneous appearance and a high reflectivity of the acinar unit walls (**B**).

For each patient, three high-quality images of various depths of the lower eyelid tissue were chosen randomly for the healthy fellow eye and the anophthalmic socket. ImageJ (open-source software program, National Institutes of Health, USA) was used as the image processing tool. The density of the MG acinar units was identified by marking acinar units manually (Fig. 1) and mean values were calculated. Acinar unit diameters were measured along the longest axis of the acinar units and acinar unit areas were measured along the inner acinar unit walls. Minimum, maximum, as well as mean values, were recorded. Secretion reflectivity and inhomogeneous appearance of acinar unit walls and the inhomogeneous appearance of the periglandular interstices were graded based on a 4-point scale developed by Villani et al. [41, 42].

### Statistical analyses

SPSS Version 26.0 for Mac (SPSS, Inc., Chicago, IL) was used for all statistical analyses. Shapiro–Wilk tests were performed to analyze the normal distribution of all scores, values, and measures of the LSCM. To compare the scores and values between the anophthalmic and fellow sides for the Schirmer test, the acinar unit diameters, acinar unit density, as well as mean and maximum acinar unit areas *t*-tests for paired samples due to normal distribution were performed, respectively. Wilcoxon tests were used to identify statistical differences for the scores of the three dry eye questionnaires, secretion reflectivity, inhomogeneous appearances of the interstices, and the acinar unit walls as well as the minimum acinar unit areas between the anophthalmic and fellow sides.

To compare the OSDI, SANDE, DEQ-5, conjunctival socket inflammation scores, and LSCM measures between enucleated, eviscerated, and not operated anophthalmic sockets Kruskal–Wallis tests and Mann–Whitney *U* tests as post hoc tests were performed, respectively.

To investigate factors related to LSCM values including acinar unit diameters, acinar unit areas, secretion reflectivity, and inhomogeneous appearance of acinar unit walls as well as the inhomogeneous appearance of the periglandular interstices general linear models were used (one for each LSCM measure) with explanatory variables. of age, gender (male vs. female), years of wearing a prosthesis, age of the current prosthesis, frequency cleaned (less than monthly, between weekly and monthly, less frequently than daily but up to and including weekly, exactly once daily, and more than once a day), the presence of environmental factors causing dry eye, as well as eyelid positions including ectropion, entropion, and lagophthalmos.

To investigate factors associated with OSDI, SANDE, DEQ-5 scores, Schirmer I test, and conjunctival socket inflammation grade general linear models were used, respectively, with explanatory variables. including acinar unit diameters, acinar unit areas, secretion reflectivity, and inhomogeneous appearance of acinar unit walls as well as the inhomogeneous appearance of the periglandular interstices.

The threshold for statistical significance was set at *p* < 0.05.

## Results

### Demographics of study population

Fifteen patients agreed to participate in the study with one patient declining due to the lack of time. Of these 15 patients, 11 were males and 4 were females. These 15 enrolled patients had a mean age of 46.2 ± 12. 7 years (range, 22–71 years) (Table 1). The right eye was lost in six cases (40%), the left eye in nine cases (60%). Reasons for eye loss included congenital (7%), medical (13%), and accident (80%). Sixty percent (60%) of patients’ eyes were enucleated, 33% eviscerated, and 7% had a microphthalmos. The mean time since the present prosthesis was fitted was 1.4 ± 0.8 years (range, 1–4 years), and the mean time since eye loss was 27.2 ± 14.9 years (range, 1–64 years).

**Table 1:** Demographics of 15 anophthalmic patients with at least 1-year experience of wearing cryolite glass prosthetic eyes.

Characteristics of 15 study participants	
Gender	
Male, *n* (%)	11 (73.3%)
Female, *n* (%)	4 (26.7%)
Ethnicity	
European, *n* (%)	8 (53.3%)
Middle East, *n* (%)	6 (40.0%)
Indian, *n* (%)	1 (6.7%)
Age (years)	
Male, mean ± SD (range)	49.58 ± 11.80 (range, 22–71)
Female, mean ± SD (range)	36.85 ± 11.25 (range, 24–52)
Duration of prosthesis wear (years)	
Male, mean ± SD (range)	26.64 ± 17.35 (range, 1–64)
Female, mean ± SD (range)	28.79 ± 4.75 (range, 23–35)
Anophthalmic side	
Right, *n* (%)	5 (40.0%)
Left, *n* (%)	9 (60.0%)
Both, *n* (%)	–
Reason for eye loss	
Accident, *n* (%)	12 (80.0%)
Medical, *n* (%)	2 (13.3%)
Congenital, *n* (%)	1 (6.7%)
Operation	
Enucleation, *n* (%)	9 (60.0 %)
Evisceration, *n* (%)	5 (33.3%)
None, *n* (%)	1 (6.7%)
Mean time since current prosthesis fitted (years)	
Male, mean ± SD (range)	1.41 ± 0.92 (range, 1–4)
Female, mean ± SD (range)	1.27 ± 0.54 (range, 1–2)

### Eyelid positions and lagophthalmos

Of the 15 anophthalmic patients, one (6.7%) had an ectropion, two (13.3%) had ptosis, and two (13.3%) had a lagophthalmos on the anophthalmic side (Table 2). There were no eyelid abnormalities on the healthy fellow sides.

**Table 2:** Dry eye symptoms, conjunctival inflammation, Schirmer I test, and eyelid abnormalities of 15 anophthalmic sockets compared to the healthy fellow eyes.

Characteristic	All anophthalmic sockets (*n* = 15)	Healthy fellow eyes (*n* = 15)	*p*
OSDI, mean ± SD (range)	12.33 ± 12.37 (range, 0.0–45.0)	4.65 ± 8.30 (range, 0.0–30.0)	0.033
DEQ-5, mean ± SD (range)	6.20 ± 3.88 (range, 0.0–15.0)	2.13 ± 3.64 (range, 0.0–12.0)	0.026
SANDE, mean ± SD (range)	27.01 ± 23.31 (range, 0.0–66.4)	7.63 ± 16.43 (range, 0.0–51.90)	0.008
Pine et al.’s Inflammation score (0–4), mean ± SD (range)	1.93 ± 0.70 (range, 1.0–3.0)	1.20 ± 0.56 (range, 0.0–2.0)	0.005
Schirmer I test with topical anesthesia, mean ± SD (range)	25.93 ± 5.91 (range, 16.0–35.0)	22.47 ± 7.50 (range, 12.0–35.0)	0.017
Lower eyelid entropion, *n* (%)	0 (0.0%)	0 (0.0%)	–
Lower eyelid ectropion, *n* (%)	1 (6.7%)	0 (0.0%)	–
*Ptosis, n* (%)	2 (13.3%)	0 (0.0%)	–
Lagophthalmos, *n* (%)	2 (13.3%)	0 (0.0%)	–

### Care and cleaning regimes

Four patients (26.7%) left their prosthesis out overnight. Ten patients (67%) washed their hands (before touching their prostheses) always, two patients (13%) mostly, and three patients (20%) sometimes or rarely. Eleven patients (73.3%) cleaned their prosthesis at least once a day, three patients (20%) less frequently than daily but up to weekly, and one patient (6.7%) never. Thirteen patients (86.7%) cleaned the prosthesis only with water, one (6.7%) with a disinfectant solution.

### Topical medication

None of the patients used topical medication at the anophthalmic socket or healthy fellow eye.

### Environmental factors relevant for dry eye symptoms and socket inflammation

Eight patients (53.3%) were exposed to at least one environmental factor in their daily life that might cause dry eye symptoms and two (13.3%) were exposed to two environmental factors. The environmental factors were: smoke (20%), dry air and draughts (13.3%), dust (20%), and air conditioners (13.3%).

### Dry eye symptoms, socket inflammation, and Schirmer I test in anophthalmic sockets compared to the fellow eye

Patients had significantly higher scores in all dry eye questionnaires and significantly higher conjunctival inflammation on the anophthalmic site compared to the healthy fellow eye (*p* < 0.05) (Table 2). In addition, the study participants had significantly higher Schirmer I test values on the anophthalmic side compared to the healthy fellow eye (*p* = 0.017). However, the mean values were in a normal range bilaterally. There were no significant differences for socket inflammation, Schirmer I test with topical anesthesia, or for dry eye symptoms between enucleated, eviscerated, and non-operated patients (*p* > 0.05, respectively). Nine patients (60%) reported mild or more severe dry anophthalmic socket complaints in at least one of the three questionnaires.

### In vivo LSCM of the MGs of the anophthalmic socket compared to the fellow eye

**Fig. 2:**
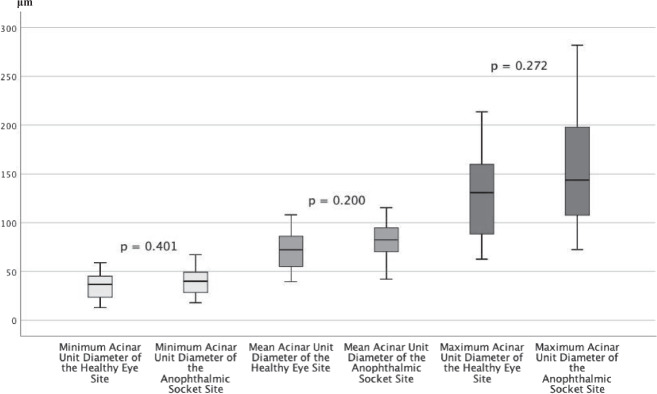
In vivo LSCM measurements of the acinar unit diameters. LSCM measurements of the acinar unit diameters of the healthy eye site compared to the anophthalmic socket site without significant differences (*p* > 0.05, respectively).

**Fig. 3:**
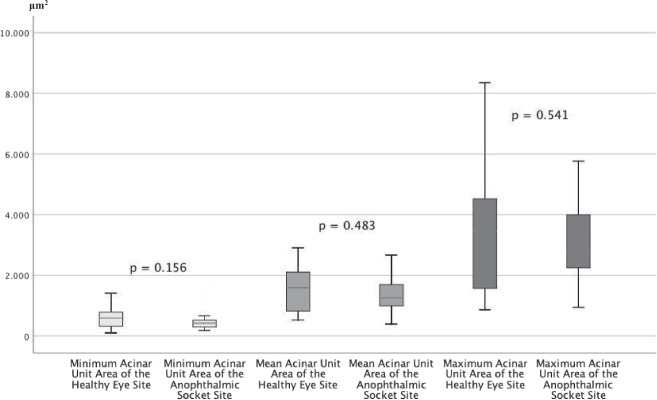
In vivo LSCM measurements of the acinar unit areas. LSCM measurements of the acinar unit areas of the healthy eye site compared to the anophthalmic socket site without significant differences (*p* > 0.05, respectively).

**Fig. 4:**
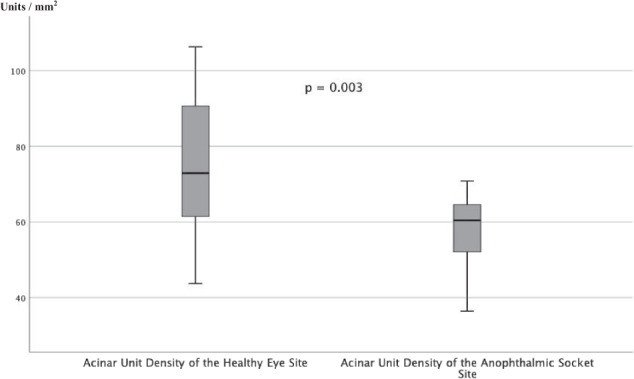
In vivo LSCM measurements of the acinar unit density. LSCM measurements of the acinar unit density of the healthy eye site compared to the anophthalmic socket site with significant differences (*p* = 0.003).

**Fig. 5:**
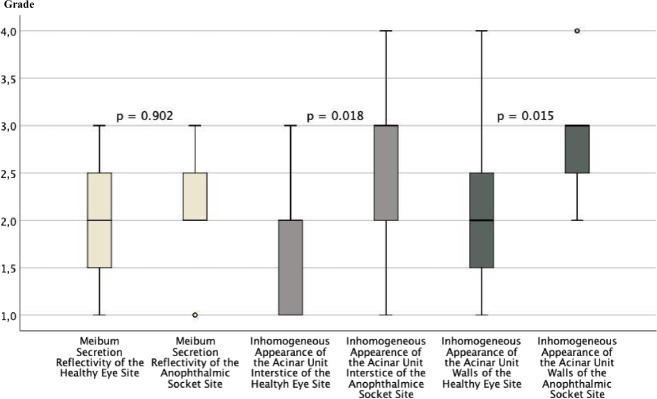
In vivo LSCM grading. LSCM grading of the meibum secretion reflectivity, inhomogeneous appearance of the acinar unit walls, and inhomogeneous appearance of acinar unit interstice of healthy eye site compared to the anophthalmic socket site with significant differences for the inhomogeneous appearance of the acinar unit walls and interstice (*p* < 0.05, respectively) but without significant differences for the meibum secretion reflectivity (*p* = 0.902).

There were no significant differences for the acinar unit diameters, acinar unit areas, and meibum secretion reflectivity for the anophthalmic socket side compared to the fellow side (Figs. 1, 2, and 3). However, there was a significant difference in the acinar unit density (Table 3) with a lower density in the lower eyelid of the anophthalmic socket (Fig. 4). In addition, there were significant differences regarding the acinar units with a more inhomogeneous appearance of the acinar unit walls and the interstice on the anophthalmic side (Fig. 5).

**Table 3:** Results of the in vivo confocal microscopy of the MGs in 15 anophthalmic sockets compared to the healthy fellow eyes.

Characteristic	All anophthalmic sockets (*n* = 15)	Healthy fellow eyes (*n* = 15)	*p*
Minimum diameter of the acinar unit (μm), mean ± SD (range)	40.00 ± 14.17 (range, 17.86–67.20)	35.51 ± 14.38 (range, 12.82–58.93)	0.401
Mean diameter of the acinar unit (μm), mean ± SD (range)	81.37 ± 19.06 (range, 42.05–115.54)	71.71 ± 21.20 (range, 39.53–107.96)	0.200
Maximum diameter of the acinar unit (μm), mean ± SD (range)	150.91 ± 60.02 (range, 72.47–281.90)	128.71 ± 48.73 (range, 62.66–213.46)	0.272
Minimum acinar unit area (μm^2^), mean ± SD (range)	466.81 ± 293.24 (range, 187.03–1385.14)	605.73 ± 383.99 (range, 101.02–1413.02)	0.156
Mean acinar unit area (μm^2^), mean ± SD (range)	1372.76 ± 673.15 (range, 392.78–2669.35)	1534.50 ± 762.00 (range, 531.08–2906.67)	0.483
Maximum acinar unit area (μm^2^), mean ± SD (range)	3114.78 ± 1571.35 (range, 926.92–5765.78)	3490.40 ± 2301.87 (range, 862.42–8350.12)	0.541
Acinar unit density (units/mm^2^), mean ± SD (range)	57.99 ± 9.74 (range, 36.42–70.83)	75.14 ± 18.92 (range, 43.75–106.25)	0.003
Meibum secretion reflectivity (grade 0–4), mean ± SD (range)	2.07 ± 0.704 (range, 1.0–3.0)	2.00 ± 0.76 (range, 1.0–3.0)	0.902
Inhomogeneous appearance of the acinar unit interstice (grade 0–4), mean ± SD (range)	2.60 ± 0.83 (range, 1.0–4.0)	1.67 ± 0.62 (range, 1.0–3.0)	0.018
Inhomogeneous appearance of the acinar unit walls (grade 0–4), mean ± SD (range)	2.93 ± 0.70 (range, 2.0–4.0)	2.07 ± 0.88 (range, 1.0–4.0)	0.015

### Factors associated with LSCM measurements

All calculated general linear models were not significant (ANOVA: >0.05 for all models). None of the investigated factors, scores, or values was associated with LSCM measures including acinar unit diameters, acinar unit areas, secretion reflectivity, the inhomogeneous appearance of acinar unit walls, or the inhomogeneous appearance of the periglandular interstices.

## Discussion

Nearly all of the study participants were very experienced and knowledgeable about wearing and handling prosthetic eyes and likely had deep insights into their problems with inflammation and especially with dry anophthalmic socket complaints. Despite the relatively small number of patients, the demographic data were very similar to the data of previous studies [3, 10, 11, 13, 35]. However, the small number is a limitation of this study as is the monocenter study design.

The higher scores of OSDI, SANDE, and DEQ-5 for the anophthalmic socket compared to the fellow eye and the finding that 60% of patients reported mild or more severe dry eye symptoms at least in one of the three questionnaires were in accordance with the results of previous studies [35]. However, since patients with clinical blepharitis were excluded from the study and previous studies have reported an association of blepharitis with higher MG dysfunction [21, 22, 29, 33, 35], the incidence of DASS is likely higher. Although 60% of all prosthetic eye wearers have at least mild dry socket symptoms, none of the study participants had a local therapy. The reasons for this remain unclear. It might be that these dry eye symptoms might be accepted by anophthalmic patients as normal in the same way that they accept mucoid discharge or perhaps there is a lack of awareness about DASS and/or of an evidence-based algorithm to treat it [1, 2, 10, 11, 13, 14, 23, 26, 35].

Our finding that patients in this study had significantly higher conjunctival inflammation on the anophthalmic side compared to the fellow eye also confirms the results of previous studies [10, 22, 35]. A longer wearing time of the current prosthesis (mean 1.4 years) over the recommended wearing time of 9 months and the frequent cleansings of the prostheses by most of the study participants can lead to higher mechanical irritation and socket inflammation [10, 22, 35]. These results suggest that prostheses should be updated on time and not be removed daily for cleaning, especially in the case of socket discomfort or socket inflammation [10, 35].

A reduced density of the MG acinar units and a more inhomogeneous appearance of the periglandular interstices and acinar unit walls in the LSCM of anophthalmic sockets without clinical signs of blepharitis might be a consequence of chronic socket inflammation. While there was no significant statistical association between the current grade of socket inflammation and morphological changes to the MGs this does not rule out the possibility that it is not the current grade of socket inflammation but rather that the duration of socket inflammation is the decisive factor. Chronic socket inflammation over long periods could lead to chronic inflammation of the periglandular interstice and of the acinar unit walls resulting in secondary loss of acinar units of the MGs, even in patients without any blepharitis signs.

That there were no significant differences for the acinar unit diameters, acinar unit areas, and meibum secretion reflectivity for the anophthalmic socket side compared with the fellow side may be a result of the exclusion of patients with any blepharitis, especially with blocked MG orifices which might have led to a changed consistency of the meibum resulting in a higher meibum secretion reflectivity in the LSCM as well as in higher acinar unit diameters and higher acinar unit areas.

Although patients had reduced acinar unit density of the MGs on the anophthalmic side and although most patients had significant dry anophthalmic socket complaints, Schirmer I tests were in a normal range bilaterally. Most previous studies have not shown a significant correlation between dry anophthalmic socket complaints and Schirmer test values [24, 26, 35, 43], suggesting that Schirmer tests may not provide sufficient diagnostic results in anophthalmic sockets [35]. The reason for this could be that there is not an absolute tear volume deficiency but rather a poor distribution of tears including a pooling in the lower fornices or behind the prosthesis [35]. The absence of a sufficient tear film over the anterior surface is likely to increase frictional resistance to blinking which in turn could add to socket inflammation. This indicates that treatment with artificial tears in these symptomatic anophthalmic patients is beneficial even in the absence of any clinical blepharitis signs.

Besides a validated and standardized questionnaire for dry anophthalmic socket complaints, ophthalmologists should evaluate patients using a standardized clinical examination protocol which includes a slit lamp examination especially with regard to conjunctival socket inflammation, anterior and posterior blepharitis, eyelid laxity, lagophthalmos, eyelid position, blink rate evaluation, and tear film break-up time. The fit and surface condition of the prosthesis should also be included while quantification of the tear meniscus and of goblet cells, evaluation of the bacterial flora, examination of the lacrimal drainage system, and MG imaging with LSCM, especially in symptomatic patients without signs of blepharitis would also be useful. The use of Schirmer tests in anophthalmic sockets is not evidence-based and further research should be undertaken in regard to this.

In summary, the majority of anophthalmic patients have significantly more dryness complaints on the anophthalmic side compared to the healthy fellow eye, even without absolute tear volume deficiency or clinical blepharitis. The DASS is a disease of the socket surface characterized by a loss of tear film homeostasis accompanied by socket discomfort, in which tear film instability, conjunctival inflammation, and damage, as well as eyelid and neurosensory abnormalities, play etiological roles [35]. Therefore, the diagnostic set of DASS should be updated to read: The presence of subjective symptoms in the anophthalmic socket are evaluated with standardized measurements (OSDI ≥ 13, SANDE ≥ 13, or DEQ-5 ≥ 6) and at least one of the five following clinical abnormalities: blepharitis anterior, blepharitis posterior, abnormalities of MGs in the in vivo confocal LSCM, reduced tear meniscus height, or conjunctival inflammation resulting in conjunctival staining. The establishment of a standardized examination protocol and treatment algorithm for DASS based on this updated diagnostic set should thus be a high priority. A standardized examination algorithm should include an evaluation of the MGs using LSCM in symptomatic patients even without signs of blepharitis or other eyelid abnormalities. Further research should be undertaken to investigate the role and the interactions of etiological causes for the DASS, especially with regard to the effect and interaction of anophthalmic socket inflammation and MG gland dysfunction.

### Summary

#### What was known before

- The dry anophthalmic socket syndrome (DASS) is a disease of the socket surface characterized by a loss of tear film homeostasis accompanied by socket discomfort, in which tear film instability, conjunctival inflammation, and damage, as well as eyelid and neurosensory abnormalities play etiological roles.

#### What this study adds

- The eyelids of anophthalmic sockets without clinical blepharitis demonstrate a reduced density of meibomian gland acinar units and a more inhomogeneous appearance of the periglandular interstices and the acinar unit walls. This might cause meibomian gland dysfunction contributing to the DASS and suggests early treatment of these patients, even in the clinical absence of any blepharitis signs.
